# Transgenerational exposure to plastics-derived endocrine‐disrupting bisphenol A and its analogs on male infertility: impact of gut dysbiosis and epigenetic regulation

**DOI:** 10.3389/fendo.2026.1799472

**Published:** 2026-05-08

**Authors:** Sanjay Basak, Saikanth Varma, Subhalakshmi Nag, Asim K. Duttaroy

**Affiliations:** 1 Molecular Biology Division, Indian Council of Medical Research (ICMR) - National Institute of Nutrition, Hyderabad, India; 2 University of Oslo, Oslo, Norway

**Keywords:** BPA, endocrine-disrupting chemicals, epigenetics, fertility, gut microbiota, gut-reproductive axis, testis

## Abstract

Global declines in male fertility, characterized by reduced sperm count, motility, and quality, raised concerns about environmental exposures to estrogen-mimicking endocrine-disrupting chemicals (EDCs), including bisphenol A (BPA), and reproductive dysfunction. BPA exposure *in vivo* has been shown to alter gut microbial composition, diversity, and metabolites, leading to dysbiosis. Such gut alterations modulate systemic inflammation, estrogen bioavailability, and the endocrine-immune axis, thereby affecting gonadal function. Even though the gut is the largest endocrine organ in the body, directly regulating multiple metabolites that reach the circulation and influence the functions of peripheral organs and systems, little is known about epigenetic perturbations due to exposure to plastic-derived endocrine-disrupting bisphenols and their role in gut dysbiosis and male infertility risks. Recent evidence on the fetal programming of bisphenol exposure suggests such events can also impact epimutation states beyond diet, potentially carrying across generations. BPA can diffuse across the membrane and enter the nucleus, altering transcription of target genes by modifying nuclear receptor activity and gene promoter methylation, similar to estradiol, a steroid hormone. The genomic imprint is modulated by gene-chemical interactions, which predominantly result in epigenetic alterations. In particular, BPA exposure *in utero* altered the epigenome, highlighting the urgent need for transgenerational assessment. This narrative review conducted a thorough review of the available data to emphasize the transgenerational impacts of BPA exposure on male infertility risk and the roles of the gut-reproductive axis, underscoring the importance of further research in this area.

## 1 Introduction

Male infertility rates have increased globally over recent decades, with declining sperm quality by count and motility reported in multiple populations (1–3). Currently, the World Health Organization (WHO) has estimated that approximately 12-17% of couples around the world are facing fertility issues, of which 30-50% issues are concerns of the male (4, 5). Growing evidence suggests that endocrine-disrupting chemicals such as bisphenol A (BPA), the highest-volume bisphenol, may contribute to these risks by disrupting testicular development, spermatogenesis, and hormonal regulation (6–8). Several studies have documented that BPA exposure alters sperm quality and causes functional defects in experimental models (9–12). While many studies have focused on direct exposure effects, fewer have examined whether BPA can induce transgenerational effects (13–16), in which reproductive impairments persist across generations even in the absence of continued exposure. Investigating transgenerational effects is particularly important because BPA exposure has been shown to alter epigenetic mechanisms, such as DNA methylation and histone modification, which can be inherited and influence gene expression in offspring (17). If BPA-induced epigenetic changes affect germ cells, particularly when exposure occurs during the developmental window, they may affect the developmental programming of male gonads and the fertility index across multiple generations, amplifying the long-term consequences of environmental exposure.

BPA is a ubiquitous plastic-derived EDC widely contaminated in food packaging (18), water bottles (19), and other consumer products. BPA is a well-established EDC that can interfere with hormonal signalling by mimicking the actions of hormones, particularly estrogenic and androgenic pathways that are critical for normal reproductive development and function (20). Human exposure to BPA is nearly ubiquitous (21), raising significant public health concerns. However, replacement of BPA with its derivatives is not as safe as previously thought, as they have been reported to possess anti-androgenic and pseudo-estrogenic effects (22, 23). These endocrine-disrupting properties of BPA affect the Leydig cell and Sertoli cells, potentially causing male infertility (24).

Epidemiological data suggest that higher urinary BPA levels are associated with a decrease in sperm concentration, along with their motility and morphology, and in addition, contribute to sperm DNA damage (25). A study claimed that seminal BPA was inversely associated with sperm count and concentration, as well as sperm morphology (26). Not only direct exposure but also gestational exposure can reduce sperm quality, mainly by impairing germ cell proliferation, the cell cycle, and androgen metabolism, leading to diminished sperm motility (27). BPA exposure has been shown to significantly reduce the viability of GC-2 spermatocyte cells in a dose-dependent manner (28). The impairment of sperm quality through perinatal exposure occurs through various mechanisms, which include steroidogenic disruption, Leydig cell apoptosis, and meiotic arrest (27). These conditions ultimately result in subfertility or infertility (27).

While the direct effects of BPA on reproductive organs have been extensively studied (29), emerging evidence suggests that BPA may also exert indirect effects by disrupting the gut-reproductive axis (30). The gut microbiota plays a critical role in regulating host metabolism (31), immune responses (32), and endocrine homeostasis (33), including the modulation of sex hormones via microbial enzymes involved in steroid metabolism (34). BPA exposure has been shown to alter gut microbial composition, diversity, and metabolites, leading to dysbiosis and increased intestinal permeability (35). Such gut alterations can influence systemic inflammation, estrogen bioavailability, and hypothalamic–pituitary–gonadal axis signaling, all of which are essential for normal reproductive function. Assessing BPA exposure within the context of the gut-reproductive axis is therefore a prerequisite for understanding the integrated, systemic mechanisms underlying BPA-induced reproductive toxicity. Despite available information on endocrine-disrupting chemicals on male reproductive functions, epigenetic impacts of transgenerational BPA exposure on testicular functions and the mediation of gut dysbiosis on male infertility risks are unknown. Evaluation of BPA effects on the gut-reproductive axis could help identify preventive strategies to protect reproductive health.

The methodology used to prepare this narrative review drew on all available scientific literature on endocrine-disrupting chemicals, BPA and its analogs, and male fertility, with a special focus on transgenerational effects. Moreover, the review proposed to elaborate on the impact of BPA on gut microbiota and its interaction with male fertility, utilizing all available data up to December 2025. The literature was collected and collated from several databases, such as PubMed, Google Scholar, and Scopus, using search terms such as “bisphenol A”, “bisphenol”, “BPA, AND male infertility” OR “transgenerational” OR “multigeneration” OR “epigenetics” OR “spermatogenesis” OR “DNA methylation” OR “Histone modifications” OR “Gut microbiota” OR “testicular microbiome” OR “autophagy”. Therefore, data from cell line, animal, and human studies examining the transgenerational impact of BPA on male fertility and testicular function, and on the gut microbiota, were considered for inclusion.

Incremental declines in male fertility could be multifactorial, including reproductive dysfunction, which may not result solely from direct endocrine disruption but also from BPA-exposure-mediated changes in gut microbiota that modify hormone metabolism and signaling pathways. Metabolites produced by gut bacteria through microbial metabolism or *de novo* synthesis affect the male reproductive system through nutrition, immunity, and hormone-related functions (36). The gut microbiota and the testis form the gut-testis axis, a bidirectional relay between gut microbes and the testes. On the one hand, modulation of the gut microbiota is beneficial in treating male infertility by enhancing spermatogenesis and improving sperm motility. Again, gut dysbiosis leads to male infertility. The concept of the microbiome-gut-testis axis underscores the complex interplay between gut health, systemic inflammation, and testicular function. Disruptions in gut or testicular microbiota can influence hormonal regulation, oxidative stress, and the integrity of the blood-testis barrier, all of which are critical for normal sperm production. Investigating this axis can provide novel insights into sex-specific susceptibility, developmental windows of vulnerability, and potential microbiota-targeted interventions to mitigate reproductive disorders. This is the first comprehensive narrative review of plastic-derived EDCs, BPA, examining their transgenerational effects on epimutation in the broader context of male fertility and their modulation of the gut-testis axis.

## 2 Transgenerational effects of BPA exposure on testicular functions

### 2.1 Impact on spermatogenesis

Spermatogenesis is a process in which haploid sperm cells are formed from diploid precursor spermatogonial germ cells in the seminiferous tubules of the adult testis. The blood-testis barrier (BTB), formed by Sertoli cells, protects developing sperm from the immune system and provides them with nutrition. The BTB is formed by tight junctions, gap junctions, ectoplasmic specializations, and desmosomes, which collectively play roles in maintaining the integrity of the BTB, which is essential for spermatogenesis and male fertility (37). Gestational exposure of ICR mice to BPA (50 mg/kg bw/day) from embryonic day 0.5 to 18.5 decreased sperm count and motility parameters, and arrested the meiotic transition from zygotene to pachytene in spermatocytes. BPA exposure also impaired spermatogenesis in male offspring by decreasing vimentin expression, a blood barrier protein (27).

Exposure to BPA (5 or 50 mg/kg/day) from 6 to 12 weeks of age to ICR male mice (F0) resulted in decreased junctional proteins mediated by binding to estrogen receptors (ERα and ERβ), disrupted spermatogenesis, and reduced sperm count with inability to fertilize eggs in the F1 generation (38). Similar effects were transferred to the successive generation (F2), partly through DNA methylation, but these effects were alleviated in the F3 males (38). Similarly, CD-1 male mice exposed to BPA (5 or 50 mg/kg/day) for 6 weeks were mated with untreated female mice to produce F1 offspring. Subsequent F2 and F3 generations were produced without exposure to BPA. Paternal BPA exposure disrupted spermatogenesis, leading to reduced sperm count and increased reactive oxygen species in F0 to F3 offspring. Changes were associated with the alterations in the global DNA methylation in the spermatozoa of the transgenerational offspring (F0 -F3) (16). Exposure of BPA via dietary administration in zebrafish has an adverse impact on reproductive tissues of their next generation and transgenerational progeny. For instance, the sperm motility of the offspring of the BPA-treated ancestral zebrafish (1 μg/g diet) was significantly lower, and the effect was evident till F2 generation, suggesting transgenerational adverse effects (39, 40). Testicular transcriptomic analysis by RNA sequencing reveals alterations in genes involved in cell adhesion, fatty acid biosynthesis, and steroid hormone biosynthesis in the BPA-treated group (41).

Like the direct exposure impact of BPA on multigenerational and transgenerational inheritance, gestational exposure to BPA is also reported to affect spermatogenesis in subsequent generations. For instance, BPA exposure (5 or 50 μg/kg/day) to pregnant female mice from gD7 to 14 impaired spermatogenesis and disrupted testicular germ cell organization in the F1 and F2 generation. Modifications in sperm DNA methylation and proteomic changes were associated with abnormal reproductive outcomes in multigenerational offspring (15, 42). Transgenerational effects of the BPA at doses of NOAEL (no observed adverse effect levels) and LOAEL (lowest observed adverse effect level) showed reduced anogenital distance, delayed onset of puberty, and disrupted testis morphology in the F0 and F1 generations. No effects were observed in the F2 and F3 generations, indicating that male infertility can be programmed due to exposure to EDCs like bisphenols during early developmental life (43). Paternal exposure to BPA can also lead to alterations in the testicular architecture and sperm parameters in the offspring. Immature male Swiss albino mice were exposed to BPA (400 μg/kg) and mated with unexposed females to produce F1 offspring, which led to increased testis weights, a decrease in sperm count and motility, and disturbed testicular histology in both direct (F0) and intergenerationally exposed (F1) males (44).

In real-life scenarios, living organisms are exposed to a diverse range of EDCs that can simultaneously disrupt endocrine balance. For instance, direct exposure to BPA and nonylphenol singly or in combination in the F0 and F1 generation significantly reduced the lipid accumulation and altered the ratio of amide I to amide II in the testicular Leydig cells of the F2 and F3 generations (45). Gestational exposure to BPA (25 μg/kg/day) along with a high-fat diet (HFD) (39 kcal%), impaired spermatogenesis due to loss of ERα expression in the round spermatids and diplotene spermatocytes exhibiting abnormal expression of protamine-1, leading to testicular atrophy (46). The combined exposure of BPA (25 μg/kg/day) with high-fat butter (39 kcal%) to the F0 Sprague-Dawley rat dams disrupted spermatogenesis in the adult male (F3 generation) by inhibiting the expression of methyl-CpG-binding domain-3 in the testis (47). A CLARITY-BPA study with prolonged exposure to BPA (2.5 to 250, 000 µg/kg/day) from gD 6 to postnatal day 90 showed little impact on the testes, as it did not detect alterations in genome-wide transcriptomics or the epigenome of rat sperm (48). The lack of detectable molecular alterations in the CLARITY-BPA rat study may be attributed to the NCTR Sprague-Dawley strain, which is insensitive to BPA due to rapid detoxification, prolonged exposure durations compared to those in all available studies with relatively shorter durations, or compensatory endocrine mechanisms. Majorities of the studies have reported adverse effects of BPA exposure during early life, from gestation to lactation, on spermatogenesis in offspring and subsequent generations, highlighting potential risks of such exposure.

### 2.2 Sperm epigenome-wide biomarkers

**Figure 1:**
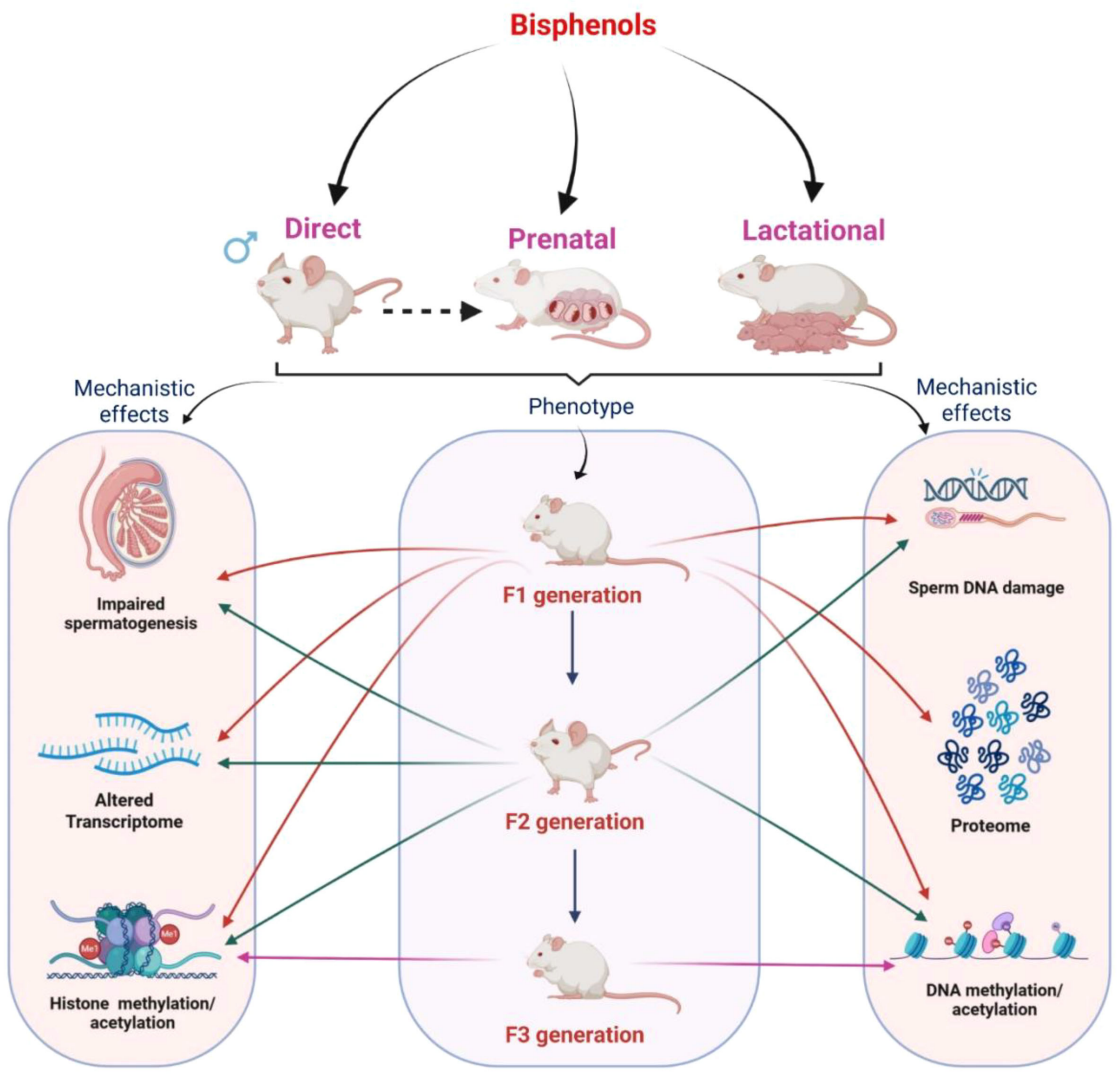
Schematic diagram illustrating the impact of bisphenol exposure (direct, prenatal, and lactational) on testicular functions in F1, F2, and F3 generations, particularly focusing on the adverse effects on epigenetic alterations observed in the male germline.

As mature sperm carry genetic material to future generations, alterations in the sperm genomic profile, caused by aberrant epigenetic modifications such as DNA methylation, can impair normal testicular development in offspring. For instance, CD-1 mice (F0) exposed to BPA and its analogs BPE or BPS (0.5 or 50 µg/kg/day) from gD 7 to birth decreased sperm concentration and motility in the F3 generation mice, which were produced from F1 and F2 offspring. Moreover, the transcript levels of Dnmt3A and Dnmt3B were increased in Sertoli and germ cells, respectively. Furthermore, H3K9me2 and H3K9me3 levels were decreased in the germ cells, indicating that BPA, bisphenol S (BPS), and bisphenol E (BPE) (0.5 or 50 µg/kg/day) induce transgenerational effects in the male reproductive tissue (49). Similarly, gestational exposure to BPA and BPS (0.4 and 4 µg/kg/day) from gD 4 to 21 increased the expression of Dnmt3A and Dnmt3B in the testis of the adult offspring, with concomitant increase in the sperm DNA methylation (23). Although many studies have shown the effect of maternal exposure to BPA on multigenerational and transgenerational male fertility, research also suggests that paternal BPA exposure (5 or 50 mg/kg) has reported alterations in the global DNA methylation of the spermatozoa of F0-F3 CD-1 male mice that were treated with BPA in the F0 generation (16). Accumulated evidence of transgenerational effects of BPA exposure on testicular functions is presented in **Figure 1**.

### 2.3 Testicular morphology, germ cell structure, and functions

The adult testis contains testicular germ cells that undergo meiosis to produce male gametes through a process called spermatogenesis. Sertoli cells in the seminiferous tubule form a BTB, dividing the seminiferous tubule into a basal and a luminal compartment. BPA can damage the BTB, leading to alterations in sperm structure and function. For instance, ICR male mice administered with BPA (5 and 50 mg/kg/day) for 6 weeks, followed by the generation of F1 to F4, resulted in seminiferous tubule and testicular abnormalities in the F0-F2 generations. However, direct exposure of BPA to the F0 male led to severe, prominent changes in spermatogonia and stemness properties of spermatogonial stem cells, which persisted through F2 but not in F3 and F4 (50). Based on several reports, the consolidated effects of transgenerational exposure to bisphenol A on fertility and male reproductive function are presented in **Table 1**.

**Table 1:** The impact of multi- and transgenerational exposure to bisphenol A and its derivatives on fertility and male reproductive functions.

Bisphenol and its derivatives	Route of exposure	Models	Sample size	Dosage	Exposure window and duration	Primary effects	Publication year	Ref
BPA	Paternal	CD-1 mice	n=40/group	5 or 50 mg/kg bw/day	6 to 12 wk	↓ levels of junctional proteins partly via ER, upregulation of p-ERK1/2, P85, p-JNK, and p38 MAPK signaling; altered spermatogenesis, ↓ sperm count, and inability to fertilize eggs in F0 to F2 males, partly by DNA methylation	2022	(38)
BPA	Paternal	Swiss albino mice	n=6/group	400 µg/kg bw	Twice per wk for 6 wk	↓ sperm count and motility, ↑ sperm abnormalities, ↑ levels of MDA, PC, and NO, and testicular damage in F0 and F1 generations	2021	(44)
BPA	Paternal	CD-1 mice	n=40/group	5 or 50 mg/kg bw/day	6 wk	Disrupted spermatogenesis, ↓ size and number of seminiferous epithelial cells, ↓ sperm motility, and count in F0-F2 males. Altered global DNA methylation in spermatozoa of F1-F3 males	2020	(16)
BPA	Paternal	CD-1 mice	n=12-15/group	5 and 50 mg/kgbw/day	6 wk	Testicular abnormalities and alterations in the seminiferous tubule, germ cells, and stemness of the spermatogonial stem cells were observed until the F2 generation	2020	(50)
BPA	Maternal	ICR mice	n=15/group	50 mg/kg bw/day	0.5 to 18.5 ED	↓ serum testosterone, testis weights, acrosomal integrity, and sperm motility; arrested meiotic transition from zygotene to pachytene spermatocytes; ↓ sperm fertility due to reduced sperm-egg binding capacity and abnormal embryonic cleavage; ↓ PCNA, vimentin, and SYCP3 expression; ↓ Leydig cell proliferation and induced G0/G1 arrest in F1 offspring	2025	(27)
BPA	Maternal	ICR mice	n=10/group	5 and 50 mg/kgbw/day	5 to 16 gD	Alterations in the sperm DNA methylation	2022	(51)
BPA	Maternal	Female mice	n=12/group	50 µg, 5 and 50 mg/kg bw/day	7 to 14 gD	F1-F2 generation- disrupted germ cell organization, spermatogenesis, ↓ sperm count, motility, and fertility; in F3 males, alterations in the sperm DNA methylation patterns and proteome changes in male germ cells of F1 to F3 generations	2021	(15)
BPA or BPS	Maternal	CD-1 mice	n=5-9/group	0.5, 50, or 1000 µg/kg bw/day	7 to 21 gD	↓ sperm counts, disrupted spermatogonia populations, elevated activity of SP1, SP4 and DMRT1 transcription factors in all F1 to F3 generations	2024	(52)
BPF	Maternal	Kunming mice	N=6/group	40, 400, and 4000 µg/kg bw/day	Gestation and lactation	Induces testicular damage in the F1 generation, ↓ T levels, and sperm quality; disrupted spermatogenesis by altering retinol metabolism, inhibited testosterone synthesis by diverting DHEA to 16α-hydroxy dehydroepiandrosterone	2025	(53)
BPAF	Maternal	ICR mice	n=14/group	300µg/kgbw/day	0.5 to 21 gD	↓ anogenital distance (AGD), testosterone levels, sperm concentration, and vitality with alterations in testicular morphology. Provoked innate and adaptive immunological responses in the testis of adult male offspring	2023	(54)
BPAF	Maternal	Sprague-Dawley rats	n=12 for vehicle control and n=14 for BPAF group	5 and 50 mg/kgbw/day	–	↓ AGD, seminiferous tubule area, germ cells, spermatocytes, Leydig cells, serum T, and FSH in F1 offspring	2022	(55)
BPAF	Maternal	Sprague-Dawley rats	n=10/group	10, 50, and 200 mg/kgbw/day	14 to 21 gD	↓ T levels, ↓ expression of Scar1, Star, Cyp17a1, Hsd17b3, and Dhh; ↓ antioxidant levels and ↑ malonaldehyde in the offspring testis	2022	(56)
BPA, BPS, and BPE	Maternal	CD-1 mice	n=6/group	0.5 or 50 µg/kgbw/day	7 to 21 gD	Disrupted progression of germ cell development, ↓ sperm motility, and concentration, dysregulated sex hormones and steroidogenesis, ↓ DNMT3A in Sertoli cells, ↑ DNMT3B, ↓ H3K9me2, and H3K9me3 levels in germ cells of F3 males	2019	(49)
BPA	Both maternal and paternal	Zebrafish	n=8/group	1 µg/g diet	3–8 months	Lower sperm velocity in F1 to F2 generations from BPA-exposed F0 parents	2022	(39)
BPS	Parental	Zebrafish	n=6/group	1 and 100 µg/L	3 hpf to 120 dfp	↑ DNA methylation led to decreased expression of steroidogenic enzymes (Cyp11a, Cyp17, and 3βHsd), ↑ E2, and ↓ T in F1 offspring	2022	(57)
BPF	Paternal	Zebrafish	–	50, 500, 2500, and 5000 nM	21 days	↓ sperm count, ↓ serum T, sperm apoptosis, and disrupted sperm meiosis in the F0 generation, whereas in the F1 generation, caused developmental issues and ↑ mortality	2025	(58)
BPAF	Paternal	Zebrafish	n=48/group	0.15 µg/L	14 days	Delayed hatchling rates, ↑ mortality, and ↑ mRNA levels of Vegfa and Cyp19a1b genes in F1 offspring	2025	(59)
BPAF	Maternal	Zebrafish	n=6/group	5, 25, and 125 μg/L	4 h to 120 dpf	↑ E2 and reduces T; ↓ egg fertilization among offspring; Induces higher malformation and lowers survival rate in the offspring	2015	(60)

Dpf, Days post fertilization; T, Testosterone; E2, Estrogen; AGD, Anogenital distance; hpf, hours post fertilization; gD, Gestation Day; ED, Embryonic day; bw, Body weight; h, hours.

“↓” indicates a decrease, and “↑” indicates an increase.

## 3 Epigenetic dysregulation of male reproductive systems due to bisphenol exposure

### 3.1 BPA exposure and its effects on transcriptomic changes

Due to its structural resemblance to estrogen, the endocrine-disrupting compound BPA can bind and activate estrogen receptors, which, in turn, bind to estrogen response elements, leading to transcriptional activation of genes. This estrogen-mimicking steroid can diffuse directly into the nucleus across the cell membrane and modulate gene transcription (61). Moreover, gestational BPA (4 μg/kg) exposure to Wistar rats for 18 days (gD 4-21) altered the offspring’s sperm energy signaling mediators in the BPA-exposed testis, due to reduced transcriptional activities of peroxisome proliferator-activated receptor gamma (PPARγ), which regulates metabolic energy demands in Sertoli cells (7). Increased PPARγ expression in ejaculated spermatozoa enhances sperm physiology, capacitation, metabolism, and viability (62). During spermatogenesis, exposure to BPA induces changes in the single-cell transcriptome in the testicular environment of adolescent males. BPA dysregulated Sertoli cell secreted proteins and enhanced the maturation and growth of germ cells (63). Transcriptomic changes encompass alterations in both the testicular and germ cells. SSCs (spermatogonial stem cells) are essential for maintaining male fertility and spermatogenesis (64). Certain specific genes and proteins, like the PCNA (proliferating cell nuclear antigen), SYCP3 (synaptonemal complex protein 3), CYP11a1 (chromosome 15q24.1), and Vimentin, have been downregulated in the offspring when exposed to BPA (50 mg/kg bw/day) in their early life (embryonic day, 0.5 to 18.5) (27). Oral administration of BPA or BPS (0.5, 50, or 1000 μg/kg/bw/day) to pregnant CD-1 females from gD 7 to birth led to a decline in sperm counts and motility in F1 to F3 adult males. Single-cell multi-omics analysis of THY1+ germ cells on postnatal day 6 from F1 to F3 males exposed to 50 μg/kg/bw/day revealed differentially expressed genes (DEGs) in spermatogonia. In spermatogonia of F1 males, 6842 and 5332 upregulated DEGs were identified in the BPA and BPS groups, respectively, compared to the control, with an overlap of 4388 genes among BPA and BPS. The DEG-linked biological processes were associated with spermatogonial meiosis, mitosis, epigenetic changes, energy metabolism, and apoptosis. The weakened biological processes are also associated with TGFβ signaling, macrophage activation, and cytokine production (65).

### 3.2 Effects of BPA exposure on DNA methylation

DNA methylation plays an important role in embryo development. Early-life exposure to BPA in developing neonates induces hypermethylation of the estrogen receptor promoter regions in the rat testis, which may be a mechanism underlying xenoestrogen-induced adverse effects on spermatogenesis and fertility (66). Neonatal exposure to BPA (2.4 μg/day) from postnatal day (PND) 1 to PND 5 in Holtzman male rats decreases sperm parameters and increases post-implantation loss due to altered methylation patterns of imprinted genes, leading to subfertility in adulthood (67). Moreover, in resorbed embryos, the expression of DNA methyltransferases (Dnmts) and related transcription factors was downregulated compared with that in viable embryos (68).

BPA, an endocrine-disrupting compound, has been shown to induce significant changes in the levels of Dnmts such as Dnmt1 and Dnmt3A, as well as global DNA methylation in the testis. It was observed that DNA methylation might play a role in regulating BPA-triggered spermatocyte toxicity (28). BPA exposure can result in both upregulation and downregulation of Dnmt1 and Dnmt3A expression, depending on species, tissue type, dose, and exposure duration. A dose-dependent study (0, 0.05, 0.5, 5, and 50 mg/kg/day) on pregnant Sprague-Dawley rats revealed that Dnmt1 and Dnmt3A were noticeably increased in the 0.05 mg/kg of BPA-exposed offspring (69). Similarly, BPA exposure to GC-2 cells has reportedly been associated with increased global DNA methylation and varied Dnmt levels (28). Gestational exposure of Wistar rats to BPA at low doses (0.4 and 4 μg/kg) starting from gestational day 4 to 21, altered testosterone levels and resulted in a dose-dependent decline in the expression of androgen receptors with upregulated expression of Dnmt3A and Dnmt3B levels in the testis of the F1 generation (23). Kunming mice exposed gestationally to BPA (40 mg/kg/bw/day) disrupted the testicular function by increasing transcription of testicular Dnmt1, whereas the Dnmt3A and Dnmt3B levels diminished in the F1 mice (70). GDNF (glial cell line-derived neurotrophic factor) mRNA and protein expression were markedly decreased in the 0.5 and 50mg/kg BPA exposure groups at prenatal day 21 (69). Methylation of the Gdnf gene promoter was significantly increased in the 0.5mg/kg BPA-exposed group, but decreased in the 5 and 50mg/kg exposed groups at postnatal day 21 (69). Epigenetic analysis of sperm showed differential DNA methylation regions (DMRs) in the F3 generation (71, 72). Embryonic exposure of medaka fish to BPA (100 μg/L) led to hyperactivation of kisspeptin and gonadotropin-releasing hormone, as well as their receptors, in the F0 generation, with global hypomethylation in the testis, and some of these effects were also observed in the F2 generation, suggesting epigenetic inheritance to subsequent generations (73). Similar findings were reported in zebrafish exposed to a low dose of BPA (0.01, 0.1 mg/L) for 15 days (74).

Genome-wide methylation status can also be assessed by the methylation of long interspersed nucleotide element (LINE-1). Studies in humans have shown that log-transformed urine BPA levels were inversely associated with sperm LINE-1 methylation levels but not with peripheral blood cell LINE-1 methylation in BPA-exposed workers compared with unexposed groups, suggesting potential risks to reproductive health in men from such exposure (75). Methylation of LINE-1, a genome-wide DNA hydroxymethyl cytosine (hmc) study was conducted on the sperm samples of men who were occupationally exposed to BPA, found that the total levels of 5-hmc were elevated significantly (19.37%) and 9, 610 differential hydroxymethylated regions, primarily in intergenic and intronic regions, affecting over 2, 000 genes compared to controls with non-occupational exposure. Moreover, about 72% of the genomic regions harbored 5-hmc. Notably, BPA altered 5-hmC patterns in ~11% of sperm-expressed genes, often in regions marked by H3 trimethylation, suggesting BPA disrupts sperm gene regulation through epigenetic mechanisms. These findings provide evidence that aberrant 5-hmC modification may contribute to BPA-related reductions in sperm quality (76). Another clinical study evaluated the effects of occupational BPA exposure on sperm acetylcholinesterase (AChE) hydroxymethylation, which is involved in sperm function and apoptosis. BPA-exposed men had significantly higher AChE 5-hmc levels than non-BPA-exposed males. A positive correlation was observed between the urinary BPA levels and AChE 5-hmc levels, suggesting that BPA may influence sperm quality by locus-specific epigenetic modifications (77). Similarly, sperm LINE-1 was found to be more hydroxymethylated in the BPA-exposed group than in the unexposed group (78).

### 3.3 Effects of BPA exposure on histone modification

Histones are the proteins present in the nucleus around which the DNA is wound to form a nucleosome. Histones can regulate transcriptional activity at promoters and genes by undergoing various modifications to amino acid residues, thereby altering the physical structure of chromatin. The modifications include acetylation, methylation, phosphorylation, and ubiquitination. Environmental estrogens, such as bisphenols, have been shown to induce epigenetic changes through histone modifications. For example, *in vitro* studies on the GC-1 spermatogonial cell line exposed to varying concentrations of BPA showed a decrease in global methylation, likely by reducing Dnmt gene expression levels. Moreover, global levels of H3K27me3 were also reduced upon BPA exposure (79). Gestational exposure (gestational day, (gD) 1-5) to BPA (20 μg/kg/day) in female ICR mice decreased the expression of StAR (steroidogenic acute regulatory protein gene) and P450scc (cholesterol desmolase) by decreasing the histone H3 and H3K14 acetylation in the promoter region of the BPA-exposed mice. Histone acetylation of the StAR promoter disrupts testicular steroidogenesis, retarding testicular development (80). Moreover, decreased sperm quality could be attributed to alterations in histone methyltransferase genes, such as Mll2-5, Setb1-2, and EZH2, upon BPA (15 μg/L^)^ exposure in adult male rare minnows (81).

Histone-to-protamine transition during spermatogenesis is a fundamental process in male fertility. BPA exposure to male mice causes abnormal histone to protamine replacement by increasing histone variants related to the histone to protamine transition in the testis (82). A recent study has demonstrated that BPA induces N6-methyladenosine RNA methylation in Leydig cells in both *in vivo* and *in vitro* (83). Transcriptomic and MeRIP (methylated RNA immunoprecipitation) sequencing data suggest that BPA exposure increases m6A levels on the Map1lc3b mRNA, a central autophagy regulator, thereby suppressing autophagy. Tet1 (ten-eleven translocation 1), a DNA demethylation marker, exhibited differential BPA-induced toxicity in TM3 Leydig cells through Cav3.3 hydroxymethylation (84). Global DNA methylation levels were elevated with a strong positive stain of H3K9me3 (histone H3 lysine 9 trimethylation) on spermatocyte, spermatid, and sperm in the adult male rare minnow exposed to BPA (15 μg/L) (81).

### 3.4 BPA exposure: a potential case for epigenetic reprogramming escape

In order for the epigenetic changes induced by the BPA to be inherited into the subsequent generations, these epigenetic marks need to escape a phenomenon called epigenetic reprogramming. Epigenetic reprogramming is a biologically fundamental process in mammals, involving erasure and remodeling of epigenetic marks such as DNA methylation, histone modifications, non-coding RNAs, and chromatin organization. The reprogramming of the epigenetic signature occurs in two steps: post-fertilization in the embryo and during the development of primordial germ cells. During spermatogenesis, DNA methylation is evenly distributed across the genome of sperm cells, spanning nearly 90 percent of it. In mature oocytes, approximately 40 percent of the DNA is methylated (85). Moreover, in addition to differential global DNA methylation percentages between male and female gametes, demethylation also differs significantly between the genomes of the two gametes. For instance, the paternal genome undergoes rapid DNA demethylation mediated by the enzymes ten-eleven translocation methylcytosine dioxygenases 1 and 2 (TET1 and TET2). However, the maternal genome does not undergo active methylation like the paternal genome; instead, it relies on passive demethylation, which occurs due to the absence of DNA methylation maintenance machinery (86). Imprinting control regions and LINE-1 elements could also resist these epigenetic reprogramming events. DNA methylation is mainly mediated by enzymes such as DNA methyltransferases (DNMTs), including DNMT1, DNMT3A, and DNMT3B, which help establish and maintain DNA methylation patterns during development and throughout life. Differential DNA methylation (DMR) patterns occur in regions that are low in repetitive elements, such as CpG islands, promoters, enhancers, and gene bodies. These regions could be potential hotspots for transgenerational epigenetic inheritance.

Several studies suggest that BPA induces epigenetic modifications that can be passed on to several generations. For instance, BPA-induced alterations in DNMT1, DNMT3A, and DNMT3B expression may compromise both the maintenance and *de novo* methylation machinery required to faithfully restore germline methylation patterns after PGC reprogramming (49). Furthermore, histone modifications such as H3K9me2/me3, reported to be disrupted by BPA, can serve as epigenetic memory scaffolds, directing DNMT3-mediated re-methylation at specific loci following PGC reprogramming (49). Neonatal exposure of male rats to BPA (2.4 µg/pup) caused significant hypomethylation of the H19 imprinting control region in the spermatozoa and in the resorbed embryos. Moreover, the transcript expression of H19 genes was reduced in resorbed embryos compared to control viable embryos. This study indicates that BPA induces aberrant methylation at the ICR in the male gamete, which is inherited by subsequent generations, implying an escape from epigenetic reprogramming (87).

The discrepancies in epigenetic outcomes observed across many studies could be attributed to the non-monotonic dose-response of BPA, which suggests that at low doses these EDCs can elicit and activate signaling pathways, whereas at higher doses they may saturate or inhibit the same pathway (88). Moreover, the DNA methylating enzyme, such as DNMTs, can be differentially sensitive to BPA concentrations (89). Additionally, the window of exposure can also lead to heterogeneous epigenetic responses.

## 4 Emerging mechanisms of BPA exposure: relevance to obesogenic effects, autophagy, and fertility

### 4.1 BPA as an obesogen induces oxidative stress that dysregulates autophagy control

Autophagy is the natural cellular process by which cells degrade and recycle their contents to maintain energy homeostasis. It is a self-preservation mechanism and an intracellular degradation system that delivers cytoplasmic contents to lysosomes (90). Dysregulated autophagy promotes obesity in multiple ways, including increased fat storage relative to fat utilization, enlarged fat cells, mitochondrial dysfunction that prevents the removal of damaged mitochondria, activation of inflammatory pathways through macrophage infiltration into adipose tissue and cytokine release, and a chronic low-grade inflammatory state. Data suggest that dysregulated autophagy promotes BPA-induced lipid accumulation in male mice (91). More specifically, dysregulated autophagy disrupts several tightly coordinated processes in the male reproductive system, especially in the testis and sperm, thereby increasing infertility risk. The dysregulation failed to maintain spermatogonia stem cell stability, the homeostasis of the meiotic cycle, and promote germ cell differentiation, leading to abnormal differentiation, apoptosis, and reduced sperm count. TM4, Sertoli cells exposed to BPA disrupted autophagy by upregulating p-mTOR and p62, an autophagic degradation protein, and decreasing Atg12 expression, an autophagy initiation protein (92). In contrast, another study using goat Sertoli cells showed that BPA decreased cell viability, increased the Bax: Bcl2 ratio, and promoted autophagy by elevating autophagosome formation (93). BPA exposure promotes autophagy in the testicular cells by activating the Akt/mTOR pathway and increasing the expression of autophagy genes and proteins (10). Prenatal and lactational exposure of BPA (50 mg/kg/day) and Di-(2-ethylhexyl) phthalate (DEHP) (30 mg/kg/day) by oral gavage to SD rats led to significant alterations in the histopathology, with elevated apoptosis and autophagy in testicular tissue (94). Prepubertal male rats exposed to BPA have also been reported to cause male reproductive toxicity by inducing apoptosis, autophagy, and ferroptosis (10, 95, 96). Another possible mechanism underlying BPA-induced spermatogenesis disorder is ferroptosis, which is associated with excessive mitophagy. BPA exposure upregulated BCAT1 expression in mice and GC-1 cells, and downregulating BCAT1 alleviated these effects (97).

### 4.2 BPA-induced autophagy and oxidative stress: p62-NRF2 and other pathways in the testis

EDC exposure during pregnancy promotes offspring with an obese phenotype. However, the BPA-induced obesity could have multiple manifestations, as obesity induces oxidative stress, which is strongly associated with infertility. Defective autophagy failed to remove damaged mitochondria, increasing reactive oxygen species (ROS), oxidative stress, and DNA fragmentation, leading to sperm DNA fragmentation. The protection of such oxidative stress is maintained by autophagy involving many pathways, such as p62/Keap1/Nrf2. In response to oxidative stress, p62 binds to Keap1, preventing Keap1 from binding the Nrf2 transcription factor. The Nrf2 translocates into the nucleus and triggers the expression of antioxidant-related genes. Thus, the p62 protein regulates the balance between damaged substrates and autophagosomes to maintain homeostasis. Since BPA exposure has obesogenic effects in offspring, and obesity is linked to abnormal autophagy activity, whether BPA-induced autophagy plays a role in male infertility needs to be examined.

*In vitro* studies using the Leydig cell line LC3 have shown that BPA (60 μM) treatment increases reactive oxygen species (ROS) production and reduces antioxidant enzyme activity. ROS triggers the opening of the mitochondrial permeability transition pore, which leaks cytochrome c from damaged mitochondria, leading to apoptosis. Moreover, the excess ROS inhibits the fusion of autophagosomes with lysosomes, impairing autophagy flux (98). BPA exposure (2 to 50 mg/kg/day) inhibits the mTOR pathway and activates the Akt pathway in the adolescent testis, leading to concurrent apoptosis and autophagy (95). Active phagocytosis was observed in the testis of the BPA-exposed group, evidenced by increased autophagosomes in the seminiferous tubule (95). The damage induced by BPA exposure was found to be reversible. Several naturally derived and pharmaceutical compounds like epigallocatechin gallate (99), resveratrol (100), lactoferrin (101), rapamycin (92), alginic acid (102), dihydroxy vitamin D3, and vitamin D receptor (103), have been shown to attenuate the BPA-induced autophagy in the reproductive tissues.

Combined exposure of BPA (5 and 50 μg/kg/day) and nonylphenol (15 and 150 μg/kg/day) exacerbated apoptosis and autophagy in SD rat prepubertal testis by increasing the expression of Beclin1, Atg5, Atg12, and LC3 protein. Moreover, apoptosis was mediated through the mitochondrial pathway, with elevated caspase-3 and Bax and a concomitant decrease in Bcl-2 protein, leading to impaired spermatogenesis (104). Intragastric administration of BPA (0.5 mg/kg) and nonylphenol (5 mg/kg) to both F0-generation male and female rats decreased sperm count and increased the LC3-II/LC3-I ratio, indicating autophagy. Additionally, impairment of the Akt/mTOR pathway was also observed in the testis of the F1 male offspring rats (105).

## 5 BPA exposure, gut microbiota dysbiosis, and male fertility: the gut-testis axis

### 5.1 BPA exposure alters gut microbiota and promotes gut dysbiosis

BPA is a plastic-derived xenoestrogen known for inducing obesogenic effects. BPA mimics a weak endocrine disruptor with documented obesogenic effects in several studies (106–109). Due to this effect, gestational exposure to BPA exhibited an obese phenotype in the F1 offspring (110). Studies in recent decades have shown that exposure to BPA is directly linked to obesity (111–113). This particular xenoestrogen has recently shown evidence supporting the fact that not only does it show obesogenic effects, but also is a leading cause of gut dysbiosis (111). Growing evidence suggests that alterations in the intestinal bacterial community due to BPA exposure can affect metabolic, systemic, and localized inflammatory responses (114, 115). Children with normal weight had a richer, more structured, and more tightly connected gut microbial network than those who were overweight or obese. Within this network’s BPA-tolerant subcommunity, greater connectivity was associated with a wider diversity of potential BPA-degrading enzymes (111). Results from a particular study also revealed that direct exposure to BPA increased the abundance of the phylum Actinomycota, whereas it reduced that of the phylum Bacteriodota. The *Clostridia* class was fully identified as BPA-resistant (116). This imbalance of the species in the intestine indicates impaired lipid metabolism and inflammation (115–117). Evidence indicates that obese subjects exposed to BPA have lower *Firmicutes* levels (26), a genus that produces butyrate, a key regulator of leptin gene expression (118). BPA exposure promotes Proteobacteria colonization and reduces the abundance of the phylum Tenericutes (119). The overgrowth of *Proteobacteria* is a key indicator of gut dysbiosis, which may be associated with inflammation (120).

The effects of BPA exposure on the gut microbiota are reported, with impairment of the natural remodeling of the gut microflora during pregnancy (115). It was observed that the genus *Dubosiella*, which is typically elevated during normal pregnancy, was absent in BPA-exposed pregnant C57BL/6 mice. Moreover, a synergistic obesogenic effect of BPA was observed when mice were fed a HFD and exposed to BPA (100 μg/kg bw/d) (115). Data suggest that BPA causes sex-dependent dysregulation of the gut microbiome. In a study where *all genders* were perinatally exposed to 50 mg/kg of BPA, the male offspring showed an abundance of *Bacteroides, Mollicutes, Prevotellaceae, Erysipelotrichaceae, Akkermansia, Methanobrevibacter, and Sutterella*, while the female offspring showed an abundance of *Lachnobacterium* spp. and *Prevotella* spp (35). Perinatal exposure to BPA combined with an HFD affects the vertical transmission of maternal gut bacteria to offspring. This disruption of microbial inheritance impairs the early development of the offspring’s gut community, increasing their risk of obesity and behavioral issues later in life (115).

The interactions between gut microbiota and EDCs can occur in multiple ways. First, the gut microbiota metabolizes xenobiotics, chemically transforming them. Second, EDCs themselves can alter the gut microbial diversity, leading to dysbiosis. For instance, exposure to BPA and ethinyl estradiol during development disrupts gut microbiota and increases *Bifidobacterium*, leading to metabolic disorders (35). Third, interfering with the enzymatic activity of the gut microbiota (121). BPA exposure simulating the human intestinal microbial ecosystem *in vitro* significantly altered the microbial community in the colon, and the percentages of microbes shared among the ascending, transverse, and descending colons increased, concomitant with a concomitant increase in gene expression associated with estrogenic effects and oxidative stress (122).

### 5.2 Gut microbiota and it’s linked with male fertility

The gut microbiota harbors a vast, diverse, and complex community of microorganisms that reside in the digestive tract and are known to exert various hormone-mimicking functions. The gut microbiota has a well-established role in digestion and immunity, but current evidence suggests roles beyond these. Obesity, a chronic metabolic state, is characterized by low-grade inflammation, in which pro-inflammatory cytokines produced in metabolic tissues can enter the bloodstream and translocate to reproductive tissues. Obesity impacts fertility potential in males by inhibiting testosterone production, negatively affecting sperm concentration, sperm DNA integrity, and sperm function (123). Moreover, in the obese model, sperm motility was significantly reduced compared with controls, with alterations in the testicular microbiota and elevated inflammatory cytokine levels, suggesting that obesity alters the testicular microbiome (124).

Mendelian randomization analyses have provided evidence of a link between the gut microbiota and the risk of male infertility. The “Gut microbiota-testis axis” describes the two-way communication between the gut and the testes, where disturbances in the gut microbiome can lead to impaired testicular function, while male reproductive hormones and factors can also influence the composition and activity of the gut microbiota (125, 126). Metabolites produced by the gut microbiota can activate aberrant immune signals and increase the production of pro-inflammatory cytokines, leading to inflammation and damage to the testicular structure.

The human testicular microenvironment was initially thought to be microbiologically sterile, but advances in sequencing technologies have revealed that the testicular microbiota harbors small amounts of bacteria, including Firmicutes, Actinobacteria, and Bacteroides. Testis microbiome analysis by ultra-deep pyrosequencing has shown that normozoospermic men have low bacterial levels in the testis, whereas individuals with idiopathic non-obstructive azoospermia have high levels of bacterial DNA, although bacterial richness is decreased, suggesting dysbiosis (127). Immature spermatozoa contain a significant abundance of Blautia, Clostridium XIVa, XIVb, XVIII, Prevotella, and Robinsoniella genera (128). Emerging data suggest that alterations in the testicular microbiome of fertile and infertile men indicate that the gonadal microbiota can affect an individual’s fertility state. Commonly, the seminal microbiota consists of bacteria belonging to these four phyla: *Actinobacteria, Bacteroidetes, Firmicutes, and Proteobacteria*. Idiopathic non-obstructive azoospermia (iNOA) individuals have shown a significant increase in testicular DNA compared with normozoospermic men (127). A study has revealed that in the testes of normozoospermic men, Actinobacteria, Bacteroidetes, Firmicutes, and Proteobacteria are the most abundant phyla, with very few detectable bacteria overall in the testis (127). A progressive rise in dysbiosis was noted in testes, escalating from those with normal sperm production to cases of iNOA where sperm retrieval was possible, and becoming most pronounced in iNOA with total absence of germ cells (127). iNOA is one of the leading causes of infertility in men, as recent evidence suggests.

### 5.3 Gut dysbiosis and its relevance to sperm motility and spermatogenesis

Gut microbiota is considered an extensive endocrine organ. In recent years, the gut microbiota has been increasingly recognized as a vital organ that plays an important role not only in metabolic, immunological, and neurological functions (129) but also in male fertility. Metabolites produced by the gut microbiota improve the motility of the sperm. For instance, *Clostridium ramosum* and *Blautia* have been reported to enhance the secretion of 5-hydroxytryptamine (5-HT) from the enterochromaffin cells. 5-HT can activate transmembrane adenylate cyclase (tmAC) and open Ca^2+^ membrane channels, activating downstream signaling pathways, causing sperm hyperactivation, which is essential for sperm motility and fertilization of the ovum (130). The DNA fragmentation index (DFI), a biomarker used to assess male fertility, is often associated with poor reproductive outcomes. An increase in specific microbial signatures, particularly *Lactobacillus iners*, contributed to a high sperm DFI (131). Metabolomics of spermatozoa from high DFI shows higher butyrate levels, which damage sperm DNA (131).

**Figure 2:**
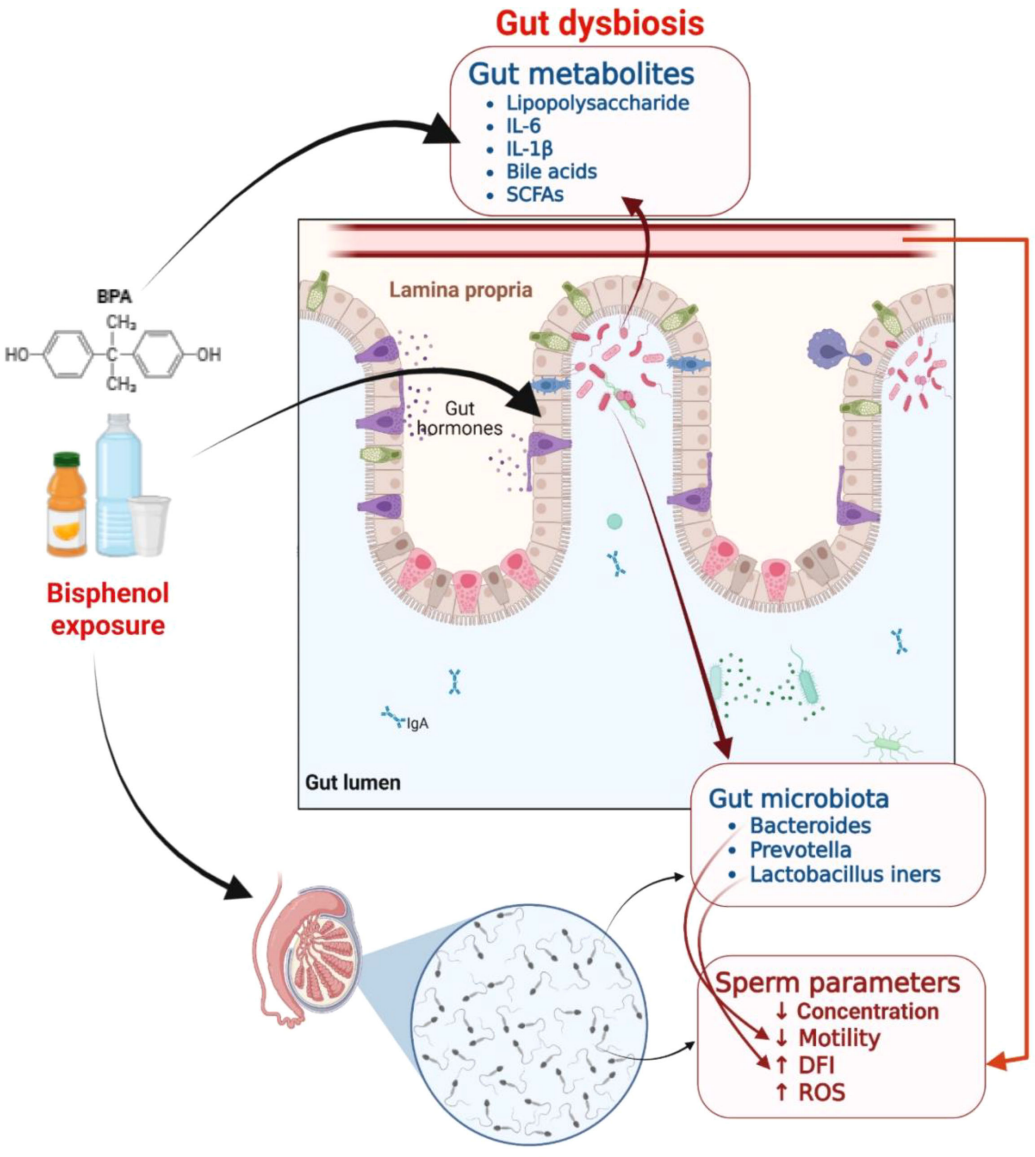
Potential modulation of bisphenol exposure on gut microbiome metabolites and testicular dysfunctions. Due to its endocrine-disrupting and oxidative stress-inducing properties, as evidenced by several *in vivo* studies, bisphenol exposure can affect intracellular junctional proteins and the blood-testis barrier, and alter the synthesis of gut microbial metabolites, thereby impairing spermatogenesis and male fertility.

Gut microbe-derived metabolites such as short-chain fatty acids (SCFAs), secondary bile acids, tryptophan/indole derivatives, and vitamins—act as pivotal signaling molecules that connect microbial community structures to sperm health and function (132). Among the SCFAs- acetate, propionate, and butyrate, butyrate is known to improve sperm count and sperm motility as observed in animal models (133, 134). Certain gut bacteria, like Lactobacillus and Bifidobacterium, are known to produce folate, which in turn regulates DNA methylation activities in fast-dividing spermatogonia (135, 136). Gut dysbiosis may alter androgen-to-estrogen ratios, thereby indirectly affecting spermatogenesis (137). A recent study showed that shifts in gut microbiota result in fluctuations in testicular polyamine levels, thereby modulating spermatogenesis (138). The effects of bisphenol exposure on gut metabolites and testicular dysfunction are emerging from several *in vivo* studies, potentially indicating adverse effects on spermatogenesis, sperm motility, fluidity, and count, as well as processes associated with male fertility (**Figure 2**).

Association studies between bacterial communities and semen quality showed that *Lactobacillus, Pseudomonas, Prevotella, and Gardnerella* are the most abundant genera in semen. The proportion of Lactobacillus and Gardnerella is reported to be higher in healthy semen, whereas *Prevotella* is abundant in inferior semen, suggesting that semen health is strongly associated with the bacterial communities (139). Research comparing the species richness and diversity of semen microbiota in fertile men versus those with abnormal semen parameters has produced inconsistent results (140). For example, some studies found fewer microbial species in infertile individuals, while others detected greater richness and diversity in the same group (140). Additionally, several studies reported no notable differences in diversity between fertile and infertile men (140). Due to these conflicting outcomes, it isn’t possible to draw definitive conclusions regarding how overall microbial diversity relates to semen health.

### 5.4 Gut dysbiosis and its impact on epigenetic modifications

The gut microbiome modulates the host epigenome through DNA methylation, systemic inflammatory response, and its fermentation products. Research suggests that the presence or absence of gut microbiota can greatly influence the host methylome. For instance, comparative studies of specific-pathogen-free, conventionally raised, and germ-free mice have shown that gut microbial colonization significantly affects the host methylome. Conventionally raised mice exhibited lower levels of TET2/3-dependent DNA methylation at regulatory elements in intestinal epithelial cells compared to germ-free mice (141).

Several studies have suggested that BPA is well known to increase the proportion of harmful bacteria and decrease the proportion of beneficial bacteria. Beneficial bacteria produce short-chain fatty acids (SCFA) like acetate, propionate, and butyrate by fermentation of carbohydrates in the colon of the large intestine. These microbial metabolites not only serve as energy sources but also act as potent signaling mediators in the gut epithelium. SCFA promotes gene expression by relaxing chromatin configuration, acting as histone deacetylase (HDAC) inhibitors (142).

**Figure 3:**
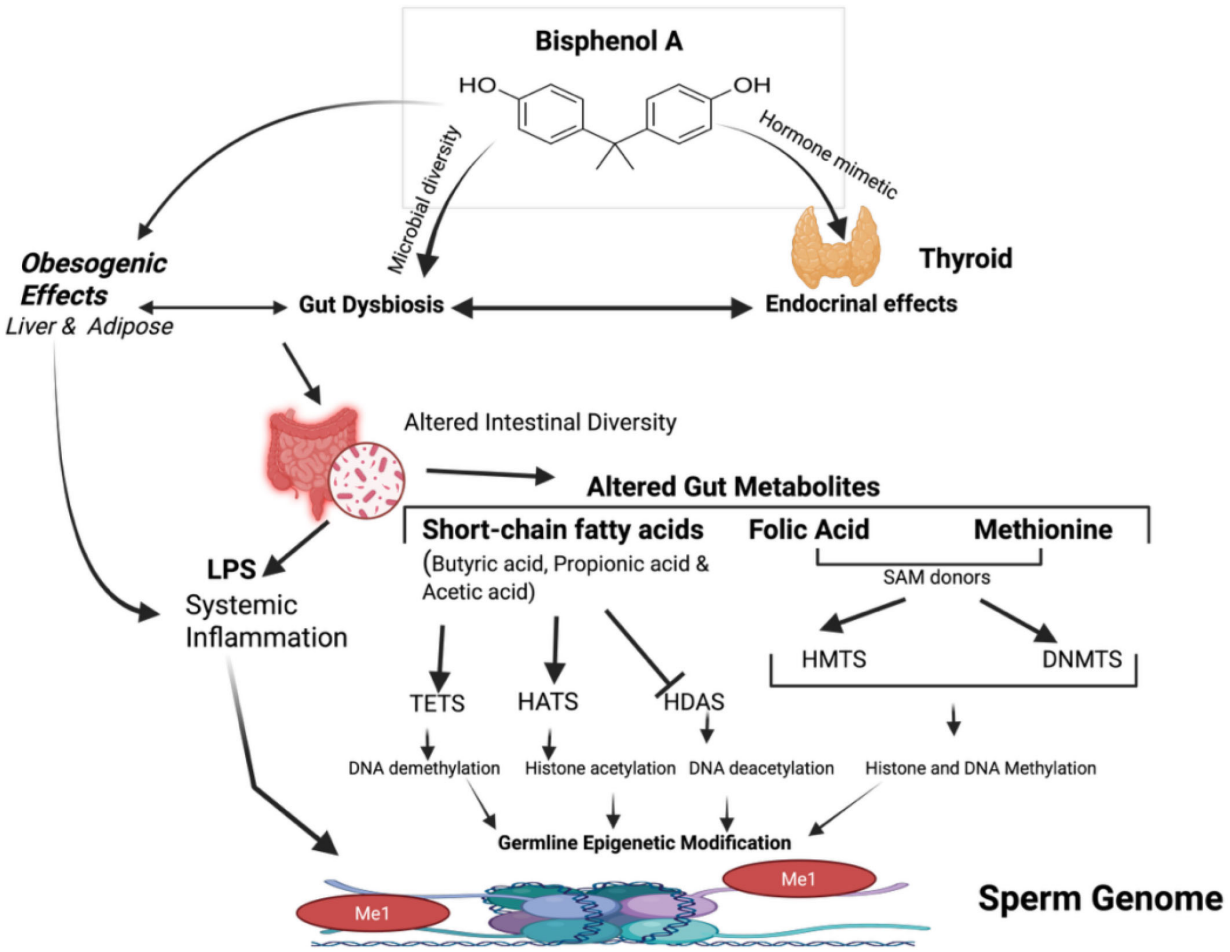
Possible mechanisms underlying crosstalk between the endocrine disruptor bisphenol A, gut microbiome, and host sperm epigenome. Overview of the epigenetic changes induced directly by BPA, causing endocrine imbalance, by acting as a hormone mimetic or indirectly through the alterations in the gut microbiota and its metabolites. BPA exposure alters the composition of gut bacteria (decreased microbial diversity), leading to gut dysbiosis. Alterations in the gut microbiota hamper its production of metabolites. Gut metabolites like short-chain fatty acids (SCFAs) inhibit histone deacetylases (HDACs) or ten-eleven translocation (TET) methylcytosine dioxygenases. Other metabolites, such as S-adenosylmethionine (SAM), serve as substrates for DNA and histone methylation enzymes, i.e., DNA methyltransferases (DNMTs) and histone methyltransferases (HMTs). Moreover, BPA can increase intestinal permeability to bacterial toxins, such as lipopolysaccharide (LPS), thereby inducing systemic inflammation and leading to epigenetic changes. Dysregulation of the epigenetic enzymatic machinery can modify the host genome’s epigenetic signature, leading to transgenerational inheritance.

Although there are no direct studies that clearly demonstrate that BPA exposure causes transgenerational epigenetic germ-line changes mediated by dysregulation of the gut microbiota or its metabolites. Based on the existing literature on gut dysbiosis and epigenetic modulations, we have now articulated a proposed mechanism as BPA induces gut dysbiosis, reducing the beneficial SCFA-producing microbiota and increasing harmful microbiota (**Figure 3**). Moreover, BPA exposure can also compromise the intestinal integrity and permeability by altering the levels of gap junctions and tight junction proteins. Decreased SCFA levels can lead to transcriptional repression, thereby reducing gene expression. Harmful bacteria produce toxins such as lipopolysaccharide (LPS), which can enter the systemic circulation through a compromised intestinal barrier. This LPS translocation can causes elicit inflammatory cytokine cascade and increase oxidative stress. Inflammation and ROS can impair the levels of DNMTs and TET enzymes in germ cells, leading to aberrant methylation changes susceptible to transgenerational inheritance.

## 6 Prebiotics, gut microbiota, and testicular dysfunction

### 6.1 Polyunsaturated fatty acids as a prebiotic candidate for sperm fluidity

Polyunsaturated fatty acids (PUFAs) in the testis are a key determinant of sperm fluidity and the male fertility index, and are also designated as a candidate prebiotic by the International Scientific Association for Probiotics and Prebiotics. Not only gut bacteria, but the metabolites produced by the gut microbiota can also modulate sperm membrane stabilization and sperm motility. PUFAs can influence testicular function and male fertility through various interconnected mechanisms and modulate the gut metabolites. Sperm membranes are rich in PUFA, especially docosahexaenoic acid (DHA), which is known to enhance membrane fluidity, motility, capacitation, and acrosome reaction (143). Gut microbes catalyze the biotransformation of PUFAs. For example, microbes such as *Bacillus proteus* and *Lactobacillus plantarus* transform the omega-3/omega-6 PUFA precursor ALA (alpha linolenic acid) and LA (linoleic acid) into their conjugated forms and further modified to form saturated fatty acid stearic acid C18:0, thereby reducing PUFA composition in the host (144). Case studies have shown that omega-3 PUFA-rich diet supplementation significantly increases microbial population involved in SCFA-producing genera (butyrate), including *Coprococcus, Bacteriodes, Blautia*, and *Roseburia* on the human gut microbiota (145). Some bacteria, such as Bifidobacterium, modulate fatty acid uptake by the intestinal epithelium, thereby directly altering the availability of omega-3 PUFAs (146).

A meta-analysis of randomized clinical trials (RCTs) showed that supplementation with DHA/EPA (Eicosapentaenoic acid) significantly increases sperm motility and seminal DHA concentration in infertile men but had no effect on sperm concentration (147). Moreover, PUFAs can also decrease oxidative stress and improve sperm function. In a randomized study on infertile men, daily DHA supplementation (0.5–2 g) for up to 3 months increased progressive sperm motility and showed a slight benefit in oxidative stress (reduced lipid peroxidation) compared to placebo (148). The ratio of n-3 to n-6 PUFA affects sperm parameters. In a cross-sectional study of men with idiopathic oligoasthenoteratozoospermia, infertile men had lower sperm and plasma levels of omega-3 FAs (DHA, EPA) and a higher omega-6/omega-3 ratio; these ratios strongly negatively correlated with sperm count, motility, and morphology (149).

### 6.2 Improvement in sperm quality and spermatogenesis: effects of probiotics supplementation and fecal microbiota transplantation

There are mainly two reported ways to target male infertility through a gut microbiota approach. One is probiotic supplementation, and the other is fecal microbiota transplantation (FMT). A study has found that changes in sperm motility in Duroc pigs are mainly due to *Rikenellaceae* (150). Probiotic supplements have also been found to positively impact spermatogenic impairment (151). Lactobacillus plays a significant role in combating male infertility (33), primarily by promoting weight loss and enhancing endogenous testosterone levels. In this regard, *L. rhamnosus strain NCDC 610* and *L. fermentum strain NCDC 400* are used as pharmaceutical alternatives and need special mention (152).

Clinical studies on asthenozoospermic males with two selected antioxidant probiotic strains (*Lactobacillus rhamnosus* CECT8361 and *Bifidobacterium longum* CECT7347) ingested for 3 and 6 weeks improved the sperm motility and reduced sperm DNA fragmentation and reactive oxygen levels, suggesting that probiotics supplementation enhances sperm motility and function (153). Another triple-blinded RCT conducted in idiopathic male infertile subjects who received a capsule (500 mg) containing a mixture of probiotic and prebiotic (*Lactobacillus rhamnosus, Lactobacillus casei, Lactobacillus bulgaricus, Lactobacillus acidophilus, Bifidobacterium breve, Bifidobacterium longum, Streptococcus thermophilus*, 10^9^ CFU), and fructooligosaccharides as a prebiotic for 80 days showed improvements in sperm morphology, concentration, motility, DNA fragmentation, and sperm lipid peroxidation compared with placebo controls (154). Although few studies have demonstrated the efficacy of these probiotics in therapeutic use to treat male infertility, well-defined RCTs with adequate subjects are required. The supplementation with probiotics regarding testicular function and fertility outcomes in human subjects is presented in **Table 2**.

**Table 2:** Associations of gut microbiota with the testicular functions and fertility outcome in male human subjects.

Study design and subjects	Population	Intervention	Dosage and duration	Primary outcomes	Ref.
Triple-blind, randomized, placebo-controlled clinical trial n=56	Idiopathic male infertility	FamiLact contains bacterial strains of *Lactobacillus rhamnosus, Lactobacillus casei, Lactobacillus bulgaricus, Lactobacillus acidophilus, Bifidobacterium breve, Bifidobacterium longum, Streptococcus thermophilus* (10^9^ CFU), and fructooligosaccharides as a prebiotic.	Single capsule of 500 mg of FamiLact for 80 days	↑ Sperm concentration, motility, and normal morphology, ↓ Sperm lipid peroxidation, and DNA fragmentation.	(154)
Randomized, double-blind controlled clinical trial n=52	Men with idiopathic oligo-asthenoteratozoospermia	Probiotics *(Lactobacillus* + *Bifidobacterium* mixture)	500 mg of probiotics daily for 10 wk	↑ Ejaculate volume, sperm number, concentration, and total motile sperm, total antioxidant capacity, ↓ oxidative stress markers (MDA), ↓ Inflammatory markers.	(155)
Double-blind randomized clinical trial n=78	Men undergoing subinguinal microscopic varicocelectomy	FamiLact contains bacterial strains of *Lactobacillus rhamnosus, Lactobacillus casei, Lactobacillus bulgaricus, Lactobacillus acidophilus, Bifidobacterium breve, Bifidobacterium longum, Streptococcus thermophilus* (10^9^ CFU), and fructooligosaccharides as a prebiotic.	2 times a day for 3 months post-surgery	↑ sperm concentration, normal morphology	(156)
male infertility (N = 733, 479), and abnormal spermatozoa (N = 209, 921)	Phenotypes of male infertility and abnormal spermatozoa	Genome-wide association study, Mendelian randomization study	–	Ruminiclostridm genus was found to be closely associated with male infertility, and the decrease in its quantity increased the risk of male infertility Decreased Prevotella was found to be closely related to abnormal sperm	(157)
N=18, 340	Data on human gut microbiota from the MiBioGen consortium and FinnGen Consortium R8 data were used to obtain GWAS data for male infertility	Two-sample Mendelian randomization (MR) study	–	Positive association between Allisonella, Anaerotruncus, Barnesiella, Intestinibacter, and Lactococcus with male infertility risk and	(158)

“↓” indicates a decrease, and “↑” indicates an increase.

For instance, the transplantation of fecal gut microbes from HFD fed mice to normal diet-fed mice led to a significant decrease in spermatogenesis and sperm motility. Genus Bacteroides and Prevotella were found to be increased, both of which cause metabolic endotoxemia in fecal-transplanted mice. Moreover, clinical studies have found positive correlations between blood endotoxin levels and Bacteroides, and negative correlations between Bacteroides and Prevotella, as well as between Bacteroides and sperm motility. Epididymal inflammation may impair sperm motility (159).

FMT, is a valuable tool for improving the gut microbiota of an individual to treat male infertility. A study showed that in bulsufan-induced male infertility, which is a common chemotherapeutic drug responsible for causing male infertility, spermatogenesis could be improved by fecal microbiota transplantation combined with alginate oligosaccharides (AOS-FMT) (160). FMT alone could also restore spermatogenesis in mouse models, although to a modest extent (161). Moreover, AOS rescued impaired spermatogenesis by supporting the gut microbiota, specifically by increasing beneficial bacterial species, such as *Bacteroidales* and *Lactobacillaceae*, and decreasing ‘harmful’ bacteria, including *Desulfovibrionaceae* (162). Similarly, fecal virome transplantation (FVT) has been shown to shape the gut microbiome. For example, FVT did not enhance colonization by administering probiotics; instead, it increased endogenous *Akkermansia muciniphila*, suggesting that the virome supports native commensals. Interestingly, FVT mice also showed unexpectedly higher fertility rate, indicating a probable link between gut virome and fertility (163).

## 7 Conclusion and future perspective

Innumerable environmental factors are known to alter gut microbiota homeostasis, among them a large number of endocrine-disrupting chemicals, such as BPA, which play a significant role. Exposure to BPA alters gut microbiota composition and function, contributing to dysbiosis through its obesogenic effects. The key mechanisms by which gut dysbiosis contributes to obesity are reduced microbial diversity, enrichment of obesity-linked taxa, and disruption of metabolic pathways. Such BPA-mediated obesogenic effects promoted gut dysbiosis and systemic inflammation, which have been shown to regulate gonadal hormone homeostasis, testicular structure, and sperm quality. In addition to direct endocrine disruption, a large body of *in vivo* data indicates that BPA-induced gut dysbiosis can influence spermatogenesis, sperm motility, and testicular function by altering gut metabolites, hormone signaling, autophagy, and oxidative stress, triggering pro-inflammatory pathways, and altering the testicular and seminal microbiome.

Available data are limited to pinpoint that BPA is a causal factor in the true transgenerational inheritance of epigenetic marks, as demonstrated by stable DMRs in F3 germ cells within functionally relevant regulatory regions, accompanied by persistent phenotypic alterations. At present, such evidence is limited; therefore, conclusions regarding causal germline epigenetic transmission and BPA exposure remain provisional. While differential DNA methylation in F3 germ cells may provide preliminary support for transgenerational epigenetic inheritance, the evidence for stable inheritance of histone modifications such as H3K9me3 or H3K27me3 is considerably more complex and remains insufficiently substantiated. Given the extensive chromatin remodeling during spermatogenesis, including the histone-protamine transition and post-fertilization reprogramming events, demonstrating persistent, locus-specific histone modifications across unexposed generations requires rigorous mechanistic evidence. In the absence of germline-specific, F3-resolved, locus-level chromatin profiling, causal inference remains premature, particularly when attempting to integrate gut dysbiosis as an upstream driver.

Nevertheless, the BPA-induced intergenerational and transgenerational exposure risks can involve the insertion of epigenetic marks, such as DNA methylation or histone modifications, in germ cells via epimutations, since BPA-induced transgenerational (F1 to F3) epigenetic alterations have been shown to affect spermatogenesis by modulating estrogenic and androgenic signaling in model systems. These long-term impacts suggest that plastic-derived EDCs like BPA can contribute to male infertility beyond immediate exposure. However, whether BPA-induced epigenetic alterations in intestinal or immune cells feed back to shape the gut microbiome composition needs to be examined further. Moreover, it is necessary to investigate whether gut microbiota-derived metabolites directly or indirectly modulate epigenetic marks in germ cells during male reproductive development. Most importantly, robust clinical data are required to support these findings and drive public health efforts to mitigate the generational burden of environmental contaminants on human fertility.
